# High-quality draft genome sequences of green microalga *Chlorella vulgaris* PKVL7422

**DOI:** 10.1128/mra.00698-24

**Published:** 2025-03-26

**Authors:** Su-Hyun Kim, Min-Jeong Kim, Tae-Jin Choi

**Affiliations:** 1 Department of Microbiology and School of Marine and Fisheries Sciences, Pukyong National University 34998, Busa, South Korea; 2 Library of Marine Samples, Korea Institute of Ocean Science & Technology, Geoje, South Korea; Rochester Institute of Technology, Rochester, New York, USA

**Keywords:** microalgae, green algae, genome

## Abstract

Despite the biotechnological interest in microalgae, there remains limited available genomic data. To contribute resources to support the genetic engineering of microalgae, a high-quality draft genome sequence was assembled for the heterotrophic freshwater microalgae *Chlorella vulgaris* PKVL7422.

## ANNOUNCEMENT

Unicellular green microalgae of the genus *Chlorella* are the most extensively studied photosynthetic organisms (1). Beijerink discovered *C. vulgaris* in 1890, making it the first microalgae with a nobly defined nucleus (2). *C. vulgaris*, which has high photosynthetic capacity and rapid growth ability under various nutritional conditions such as autotrophy, mixotrophy, and heterotrophy, is widely distributed in freshwater, marine, and terrestrial environments (3).

For genomic DNA isolation, *C. vulgaris* PKVL7422 (deposited in the Korean Collection for Type Culture, 13361 BP), isolated from a pond located at Pukyong National University in the Republic of Korea, was cultivated in BG-11 medium for 1 week at 20°C under constant illumination (60 µmol photons m^−2^s^−1^). Genomic DNA and total RNA were extracted using the Wizard HMW DNA Extraction Kit (Promega, WI, USA) and the RBC HiYield Total RNA Mini Kit for Plant (Real Biotech Corporation, Taipei, Taiwan) following the manufacturer’s instructions without modification. The DNA library was prepared using PacBio SMRTbell prep kit 3.0 (Illumina Inc., CA, USA) and Illumina Nextera DNA Flex Library Prep Kit (Illumina Inc., CA, USA) following the manufacturer’s instructions. The RNA library was prepared using the Illumina TruSeq Stranded mRNA Sample Prep Kit (Illumina Inc., CA, USA).

Sequencing data were generated through the PacBio Sequel IIe system and the Illumina NovaSeq6000 platform by Macrogen (Seoul, Republic of Korea), respectively. A total of 179,330 HiFi reads (959,995,393 bases) were produced by PacBio sequencing, and the N50 value and the average quality were 5,836 bp and Q36, respectively. Illumina sequencing generated 26,252,864 filtered reads (3,961,814,613 bases) of 42,955,062 raw data sets (6,486,214,362 bases), and the Q20 and Q30 values were 99.25% and 96.66%, respectively. The genome assembly was performed using SMRTlink 11.0.0.146107. The assembly result was corrected thrice using the high-quality adapter-trimmed Illumina reads through Pilon v1.21 to improve assembly quality (4).

For gene prediction, genome guide assembly was conducted using Illumina RNA sequencing data, which was generated from Macrogen (Seoul, Republic of Korea) using the Illumina NovaSeq platform (Illumina Inc., CA, USA). RNA reads were mapped to assembled DNA sequences using Tophat v2.1.1 (intron length set to 25–500 nucleotides) to get genome-guided transcriptome assembly data (5). RepeatMasker 4.1.2 was used for genome masking before annotation, and Maker 2.31.83 with default parameter was used to predict the gene model using the gene training model resulting from GlimmerHMM 3.0.4, AUSTUS 3.3.2, and SNAP 2.31.83 based on *Chlorellaceae* proteins downloaded from the National Center for Biotechnology Information (NCBI) in March 2023 (6–8). Predicted protein sets were subjected to InterProScan v5.30-69.0 and psiblast v2.4.0 with EggNOG DB v4.5 (9–11).

**Fig 1:**
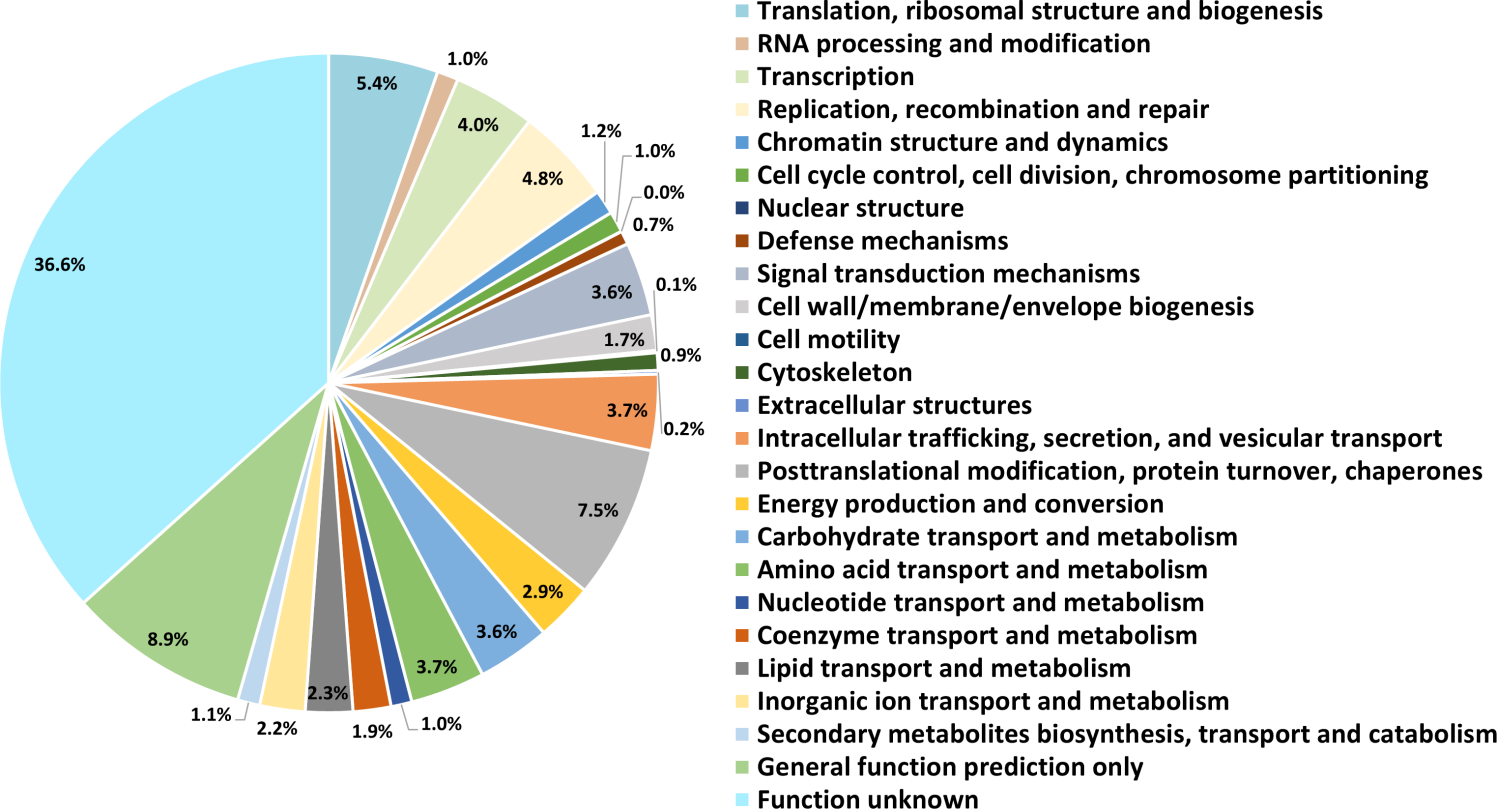
eggNOG classification of gene predicted from *C. vulgaris* PKVL7422. The proportion of eggNOG categories of 6,428 putative protein-coding genes is depicted with the categories.

The assembled genome covered approximately 23.5 times the genome size of *C. vulgaris* PKVL7422, with a total of 39.0 Mbp belonging to 93 contigs including circular forms of chloroplasts and mitochondria. The G + C content of 61.5%, and the N50 value of 1,011,039, with maximum, minimum, and mean lengths were 2,628,967 bp, 5,074 bp, and 439,841 bp, respectively. Among the 7,665 transcripts, 6,428 putative protein-coding genes were predicted, reflecting the detection of alternative splicing events and isoforms as annotated by MAKER (Fig. 1).

Since 2018, five assembled genomes of *C. vulgaris* have been deposited in the NCBI database, providing valuable foundational resources for genomic studies. However, four of these assemblies have limitations, including a high number of contigs (ranging from 753 to 3,810) and the absence of protein annotations. Our sequencing data, comprising 93 contigs with annotations, provide an improved genomic resource to understand *C. vulgaris*.

## Data Availability

The draft genome was deposited in GenBank under the accession number JBAHYI000000000 (BioProject accession number PRJNA1076344, BioSample accession number SAMN39938755, and the raw data set of PacBio and Illumina SRA accession numbers SRX23617876 and SRX23617877, respectively).
